# Evaluation of characteristic of human turbinate derived mesenchymal stem cells cultured in the serum free media

**DOI:** 10.1371/journal.pone.0186249

**Published:** 2017-10-19

**Authors:** Se Hwan Hwang, WeonSun Lee, Sang Hi Park, Hee Jin Lee, Sun Hwa Park, Dong Chang Lee, Mi Hyun Lim, Sang A. Back, Byeong Gon Yun, Jung Ho Jeun, Jung Yeon Lim, Jun Myung Kang, Sung Won Kim

**Affiliations:** 1 Department of Otolaryngology-Head and Neck Surgery, College of Medicine, The Catholic University of Korea, Seoul, Korea; 2 Institute of Clinical Medicine Research, College of Medicine, Catholic University of Korea, Seoul, Korea; 3 Department of biomedical science, College of Medicine, The Catholic University of Korea, Seoul, Korea; Instituto Butantan, BRAZIL

## Abstract

We evaluated the effect of serum-free and xeno-cultivation (SFXFM) on the characterization, proliferation, and differentiation properties of human nasal stem cells (airway tissue; hTMSCs). hTMSCs were isolated from 10 patients, after which patient samples were separated into two groups, an SFXFM group and a control group. The control group was treated with bovine serum-containing medium. FACS analysis revealed that SFXFM-cultured hTMSCs maintained a characteristic mesenchymal stem cell phenotype. hTMSC proliferation was not influenced by SFXFM. In addition, upregulation of IL-8 and GM-CSF and downregulation of RANTES expression were shown in response to SFXFM. Moreover, two-lineage differentiation properties (osteocyte and adipocyte) of hTMSCs were enhanced under SFXFM. Finally, the genetic stability of SFXFM-cultured hTMSCs was demonstrated by normal karyotype results. SFXFM enables good expansion, multipotentiality, and normal genotype maintenance of MSCs. Moreover, this approach serves as a substitute to conventional media for the cultivation of capable MSCs for upcoming medical applications.

## Introduction

Mesenchymal stem cells (MSCs) are a kind of adult stem cells with the potential to differentiate into various cells lineages [1], which are known to be isolated from various tissues of adults [2]. Recently, the nasal mucosa, especially inferior turbinate, was verified as an appealing origin of adult stem cells because of its unique properties. In previous studies, MSCs were isolated from human inferior turbinates (human nasal inferior turbinate derived mesenchymal stem cells; hTMSCs) discarded during simple surgical procedures [3]. Multiple studies have demonstrated that these hTMSCs exhibit differences in proliferation, differentiation, immunomodulation, cell passage, and donor age compared with MSCs originated from other sources [3–5].

Traditionally, MSCs have been expanded in vitro under fetal bovine serum (FBS) [6,7]. However, the application of animal-derived products may meet important restriction, such as transmission of xeno-antigens and infectious materials [8]. Because of these problems, alternative animal product-free media formulations such as allogeneic or autologous human serum, as well as human serum-free culture media, have been evaluated [7,9]. Recently, many studies have evaluated the effects of animal product-free media on proliferation, differentiation, or the immunomodulation properties of human bone marrow or adipose tissue-derived MSCs [10]. In the present study, we assessed the influence of serum-free and xeno-free media (SFXFM) on hTMSC characteristics and compared them to those of traditionally cultured hTMSCs.

## Materials and methods

We accomplished this study and followed the Institutional Review Board of the Catholic Medical Center Clinical Research Coordinating Center (HC15TISI0022), informed consent regulations, and the Declaration of Helsinki. We explained to all participants before the enrollment and received informed consents from enrolled patients directly before surgery. The Institutional Review Board of the Catholic Medical Center Clinical Research Coordinating Center approved our study and admitted all procedures in this study.

### Donors and cell isolation

Nasal tissues were obtained from 10 patients (all older than 20 years) undergoing partial turbinectomy. The hTMSCs derived from the same patients were assigned to both the SFXFM group and the control group and the cells from both groups were measured to compare the characteristics and potency of MSCs. Briefly, tissue samples were washed three to five times with antimicrobial liquid (Gibco, Gaithersburg, MD). Then they were rinsed twice with phosphate-buffered saline (PBS). The washed samples were sliced into 1 mm^3^ pieces. The pieces were moved into a culture dish and then veiled with a cover slide. In the SFXFM group, the culture dishes were applied with CELLstart CTS Attachment Substrate (Gibco) according to the manufacturer’s protocol. Next, StemPro^®^ MSC SFM XenoFree (Gibco) media supplemented with 200 mM L-glutamine (Gibco) was added, after which the tissue samples were cultivated at 37°C in a 5% CO_2_ atmosphere. The glass cover slide was removed after 3 weeks. The adherent hTMSCs were detached using TryplE Select 10X (Gibco). In the control group, non-coated culture dishes were used, and the explants were cultured in DMEM (Dulbecco’s Modified Eagle Medium, Gibco) containing 10% fetal bovine serum (FBS). For subculturing, 0.25% trypsin in 1 mM EDTA was used to separate the cells from the dishes. With the exception of the media and the cell dissociation agent, the culture procedures of the two groups were identical. At passage four, the hTMSCs were examined for culture media-related changes with respect to MSCs characteristics. The measurements for the cells were performed three times on all individual MSC donor pools. In addition, the hTMSCs at passages three and six were examined to check the karyotype abnormality under SFXFM cultivation.

### hTMSC cell surface marker characterization

Flow cytometry was conducted to measure the cell surface markers of the hTMSCs. To this end, hTMSCs were distributed into test tubes (BD, Franklin Lakes, NJ) with 1 x 10^5^ cells/ml. Cells were then cultured with primary antibodies for 40 min. Saturating concentrations of monoclonal anti-CD14 (all anti-human CD antibodies were obtained from BD Biosciences, San Jose, CA), CD19, CD34, CD73, CD90, CD105 and HLA-DR antibodies were used. Next, the cells were rinsed three times in buffer and centrifuged at 1200 rpm for 5 minutes. Cells were then suspended again in PBS and cultured with the appropriate 2^nd^ antibodies for 30 min in the dark at 4°C. Cell fluorescence was measured on a FACS Calibur instrument (BD), and the data were analyzed using Cell Quest software (BD).

### Cytokine assays

hTMSCs were placed with 1x10^5^ cells per well in 24-well plates and allowed to adhere overnight. The medium was gathered after 2 days, after which the levels of IL (interleukin)-1α, IL-1β, IL-4, IL-6, IL-8, IL-10, IL-12, IP-10 (CXCL10), RANTES (CCL5), TNF-a, GM-CSF, and IFN-γ were assayed with a MILLIPLEX MAP human cytokine/chemokine multiplex immunoassay kit (Millipore, Billerica, MA).

### Proliferation assay

hTMSCs were placed concurrently into 96-well tissue culture plates with 1500 cells per well. The medium was changed every 48 h. Cell proliferation was assayed with a (CCK)-8 cell counting kit (Dojindo Laboratories, Kumamoto, Japan). Briefly, the culture medium was discarded and 100 μL of new medium including 10 μL CCK-8 was applied to each well. The cells were then culured at 37°C for 4 h. Cell viability was measured for 7 days. The resultant optical density values at a test wavelength of 490 nm and a reference wavelength of 630 nm were determined at least in triplicate. A reagent blank was used as a control.

### Multi-lineage differentiation potential of hTMSCs

hTMSCs in the control and serum-free groups were placed into 12-well plates (2 × 10^5^ cells/well) containing StemPro^®^ Osteogenesis, Adipogenesis, or Chondrogenesis Differentiation media (Gibco) following the manufacturer’s protocol. Cells were allowed to differentiate for 14 days. During this incubation, medium were changed every 3 days. After 14 days, the cultures were measured for differentiation with tri-lineage specific stains. Specifically, for identifying adipogenic differentiation, the cells were examined with Oil Red O stain. For chondrogenic differentiation, the cells were gathered and assessed by toluidine blue stain. For osteogenesis, cells were examined with alkaline phosphatase stain. In addition, the expression of differentiation-specific genes was assessed by RT-PCR.

### RNA extraction from hTMSCs and RT-PCR

RNA was gathered from cells under chondrogenic, osteogenic, and adipogenic differentiation cultivation using an RNeasy Mini Kit (QIAGEN, Valencia, CA, USA). Treatment with deoxyribonuclease 1 (QIAGEN) was performed to clear genomic DNA, and 1 μg purified RNA was reverse transcribed into first-strand complementary DNA with an iScript reverse transcription kit (Bio-Rad Laboratories, Hercules, CA, USA). Of note, this kit also contains a genomic DNA elimination step (QIAGEN). Real-time polymerase chain reaction (RT-PCR) amplification and relative quantification of osteocalcin, type I collagen, Runt-related transcription factor 2 (Runx2), peroxisome proliferator activated receptor r (PPARr), AcylCoA synthetase (ACS), type II collagen, and aggrecan expression was conducted with TaqMan gene expression assays (Applied Biosystems, Foster City, CA, USA) on a Lightcycler 480 PCR system (Roche, Mannheim, Germany). All reactions were conducted in triplicate in a 20 μL volume with TaqMan probe Master Mix (Roche); 10 ng complementary DNA was applied in each reaction. Glyceraldehyde 3-phosphate dehydrogenase was used as an endogenous control. Results were assessed using Lightcycler 480 software version 1.2 (Roche).

### Karyotype analysis

Karyotypes were analyzed by inducing metaphase spreads of cells at passage 3 and 6 cultured under serum-free media. The cultures were treated with colchicine, after which they were harvested, briefly treated with a hypotonic solution, and fixed. The fixed cells were then placed into a slide, dried, and stained to identify a G banding pattern.

### Statistical analysis

R statistical software (R Foundation for Statistical Computing, Vienna, Austria) was used for statistical analyses. The significant discrepancies between groups were statistically decided with the t-test and one-way analysis of variance (ANOVA). A *p*-value <0.05 was considered to show significance.

## Results

### Characterization of hTMSCs cultured in serum-free and xeno-free medium by flow cytometry and comparison with those in the control group

**Fig 1:**
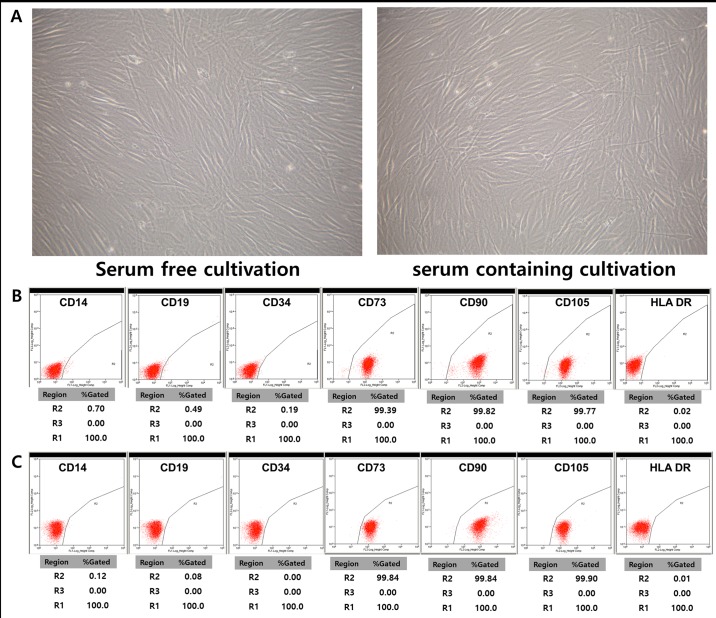
Morphology (A) after primary explant culture and fluorescence-activated cell sorting analysis of human nasal inferior turbinate derived mesenchymal stem cells (hTMSCs) cultured under serum-free medium (B) and serum-containing medium (C). Cells in both groups attached to the culture dish and displayed a similar spindle-shaped, fibroblast-like shape (magnification x 100) (A). Flow cytometry analysis revealed that hTMSCs from both groups were positive for MSC markers (CD73, CD90, and CD105) and negative for hematopoietic cell markers (CD14, CD19, CD34, and HLA-DR) (B and C).

hTMSCs in both groups showed negativity for typical hematopoietic markers (CD14, CD19, CD34, and HLA-DR) and positivity for MSC markers (CD73, CD90, and CD105) (Fig 1). This result is consistent with the phenotypical feature of MSCs

### Cytokine and chemokine secretion patterns of hTMSCs cultured in serum-free and xeno-free medium as characterized by enzyme-linked immunosorbent assay and comparison with those in the control group

**Fig 2:**
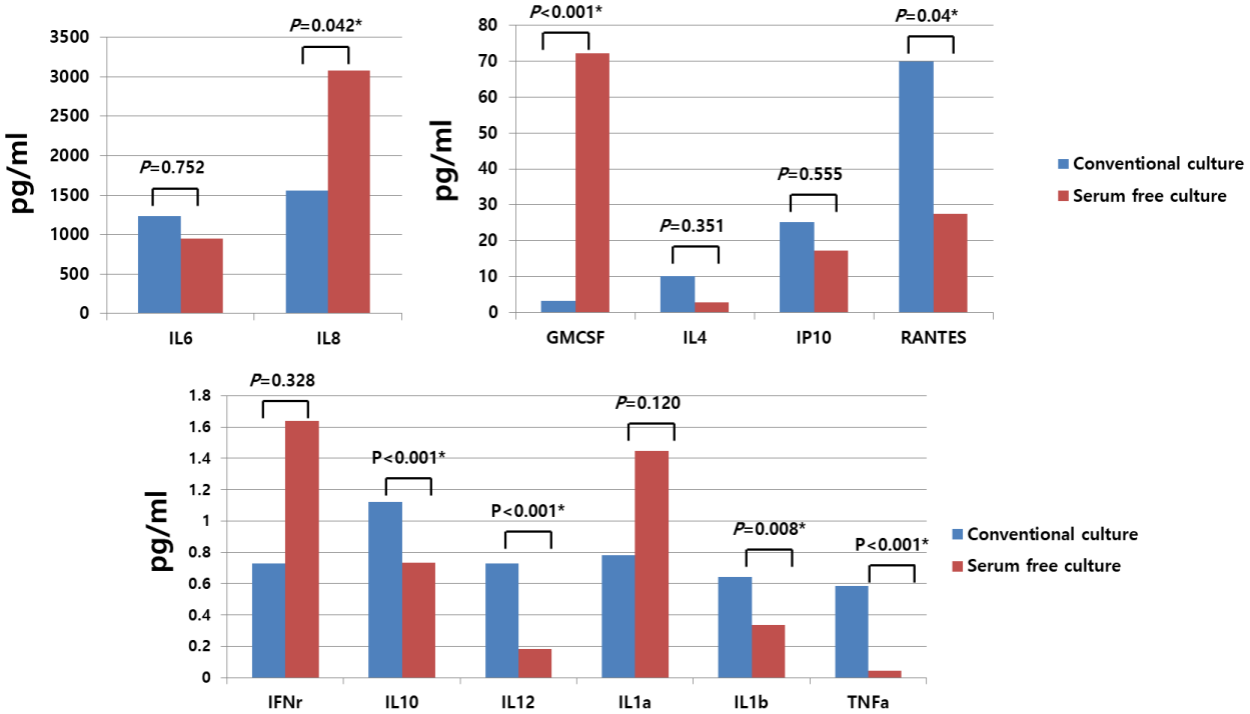
Effects of serum-free cultivation on cytokine and chemokine release by hTMSCs. Release of cytokines and chemokines [IL-1α, IL-1β, IL-4, IL-6, IL-8, IL-10, IL-12, IP-10 (CXCL10), RANTES (CCL5), TNF-α, GM-CSF, and IFN-γ] from hTMSCs cultured under serum-free medium compared to serum-containing medium was evaluated with an enzyme-linked immunosorbent assay. hTMSCs cultured in serum-free medium exhibited upregulation of IL-8 and GM-CSF, but downregulation of IL-1β, IL-10, IL-12, RANTES, and TNF-α versus the control group. These patterns of cytokine and chemokine secretion were different from those of hTMSCs cultured under serum-containing medium.

We measured the levels of a dozen of cytokines and chemokines related to immunomodulation, containing IL-1α, IL-1β, IL-4, IL-6, IL-8, IL-10, IL-12, IP-10 (CXCL10), RANTES (CCL5), TNF-α, GM-CSF, and IFN-γ. Of these cytokines and chemokines, only IL-4, IL-6, IL-8, IP-10 (CXCL10), GM-CSF, and RANTES (CCL5) were perceptible (mean value greater than 1 pg/ml) in supernatants in both groups. However, many cytokines and chemokines showed significantly different levels of secretion in the SFXFM group versus the control group. SFXFM strongly induced the expression of IL-8 and GM-CSF. In contrast, the secretion of IL-1β, IL-10, IL-12, RANTES, and TNF-α was significantly suppressed in the SFXFM group versus the control group. Specifically, an approximately 5- to 20-fold difference in TNF-α and GM-CSF secretion was observed with serum-free culture. IL-10, IL-12, IL-1β, RANTES, and IL-8 expression showed 1.3- to 2-fold differences between the groups (Fig 2). Overall, these results suggest that hTMSCs are immunologically influenced by the culture media.

### Proliferation of hTMSCs cultured in serum-free and xeno-free medium and comparison with that of cells in the control group

**Fig 3:**
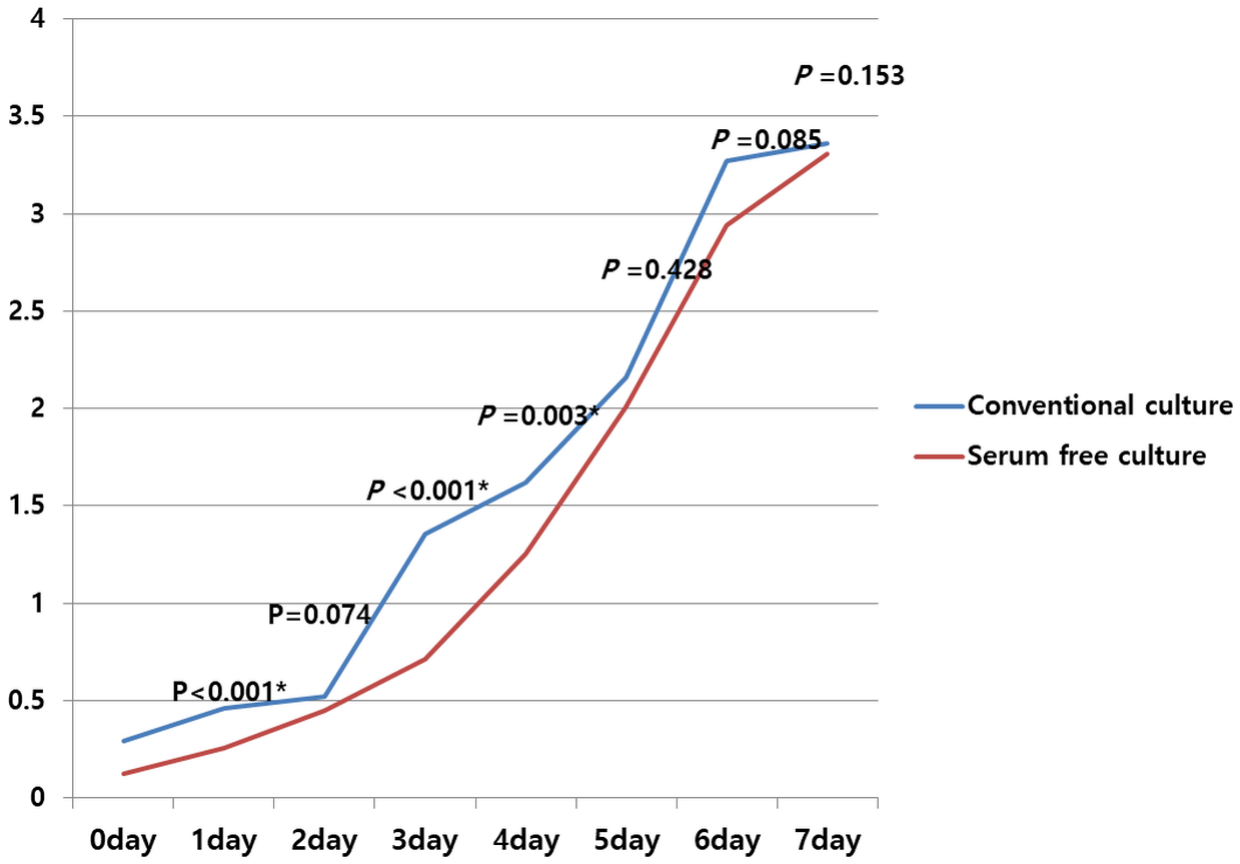
Comparison of proliferation of hTMSCs cultured in serum-free medium versus that of hTMSCs cultured under serum-containing medium. A cellular proliferation assay was conducted for 7 days. hTMSCs cultured in serum-free medium showed rapid proliferation from 3 to 4 days. The proliferation patterns resembled those observed in MSCs cultured under serum-containing medium. Additionally, culture under serum-free medium did not affect the proliferation of hTMSCs.

Cell proliferation was measured for 7 days. From days 2 to 5, the cells demonstrated fast growth. After this phase, cells then became a lag phase. The patterns of proliferation in the SFXFM group resembled those of MSCs in the control group (Fig 3). No significant differences in proliferation rate were shown in the two groups over the 7 days, although the proliferation rates were higher in the control group than the serum-free group on days 3 and 4. These results show that the cells grew consistently, regardless of the culture media.

### Tri-lineage differentiation potential of hTMSCs under serum-free and xeno-free medium as assessed by histology and RT-PCR and comparison with that of those in the control group

**Fig 4:**
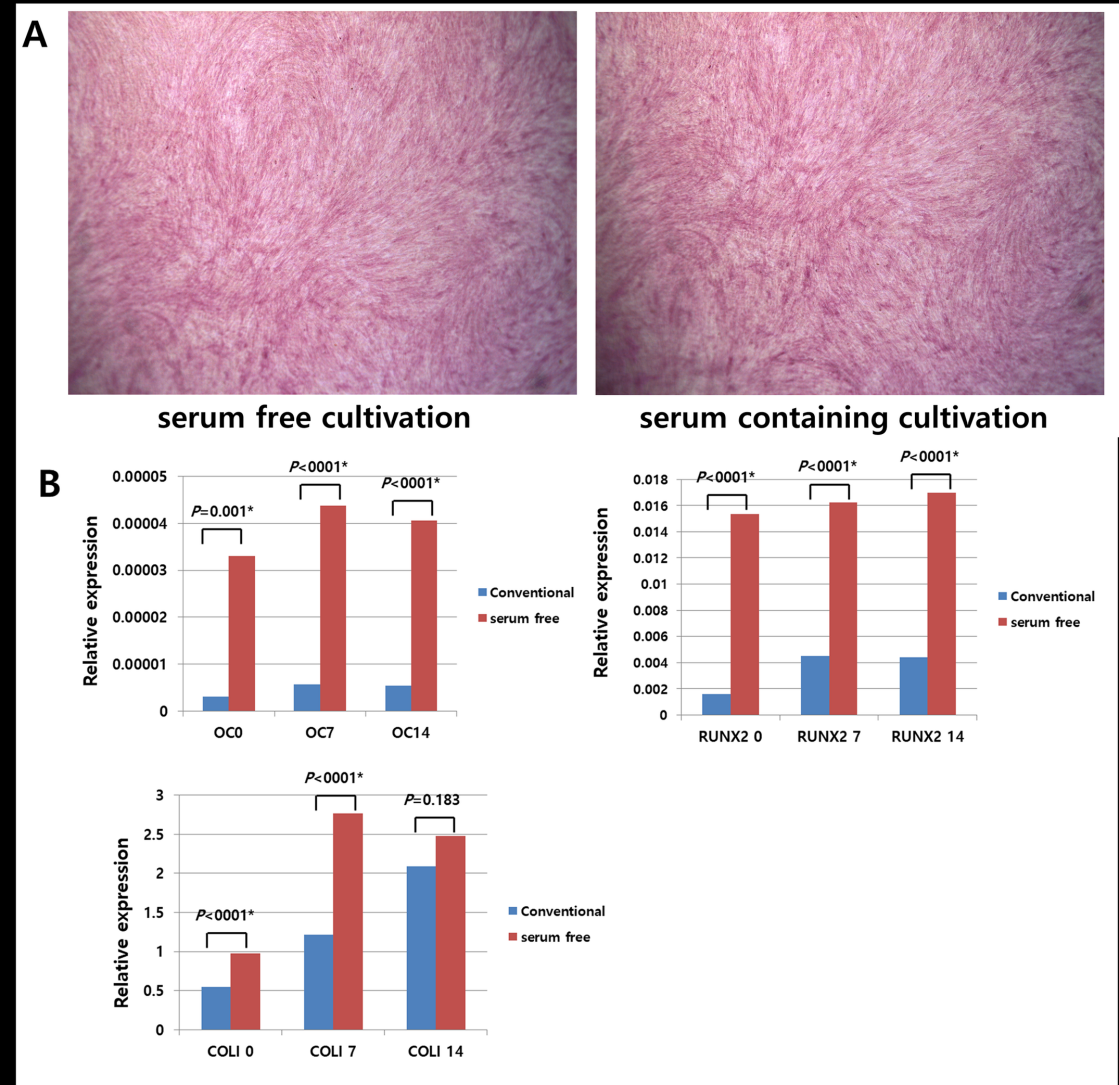
Comparison of the osteogenic differentiation potentials of hTMSCs cultured under serum-free medium versus those of hTMSCs cultured under serum-containing medium. Under osteogenic conditions, alkaline phosphatase staining (x 400) of hTMSCs cultured in serum-free medium (left) and serum-containing medium (right) demonstrated similar levels of alkaline phosphatase expression, as assessed visually. The mRNA expression levels of osteocalcin, Runt-related transcription factor 2, and type I collagen in hTMSCs (B) were perceived by RT-PCR. hTMSCs cultured under serum-free medium showed higher expression of osteogenic differentiation markers versus hTMSCs cultured under serum-containing medium.

**Fig 5:**
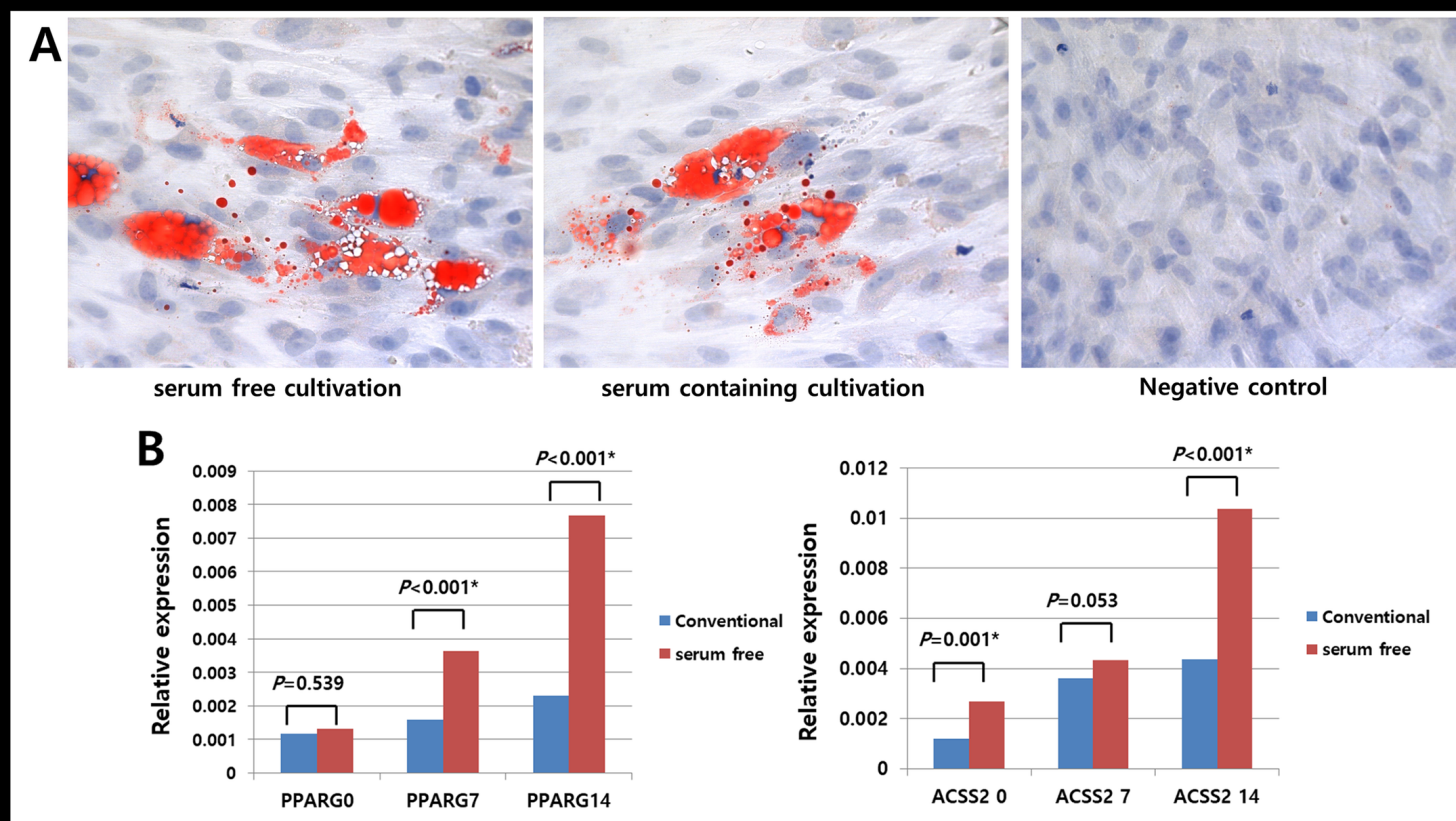
Comparison of the adipogenic differentiation potentials of hTMSCs cultured in serum-free medium versus those of hTMSCs cultured in serum-containing medium. Under adipogenic conditions, adipogenesis was perceived after 2 weeks of culture by staining of intracytoplasmic microvacuole with Oil Red O. Visual assessment revealed similar levels of intracytoplasmic Oil Red O staining (x 400) in hTMSCs cultured under serum-free medium (left) and in hTMSCs cultured under serum-containing medium (right). The mRNA expression levels of peroxisome proliferator activated receptor r (PPARr) and AcylCoA synthetase (ACS) in hTMSCs (B) were detected by RT-PCR. hTMSCs cultured under serum-free medium exhibited higher expression of adipogenic differentiation markers compared with hTMSCs cultured under serum-containing medium.

**Fig 6:**
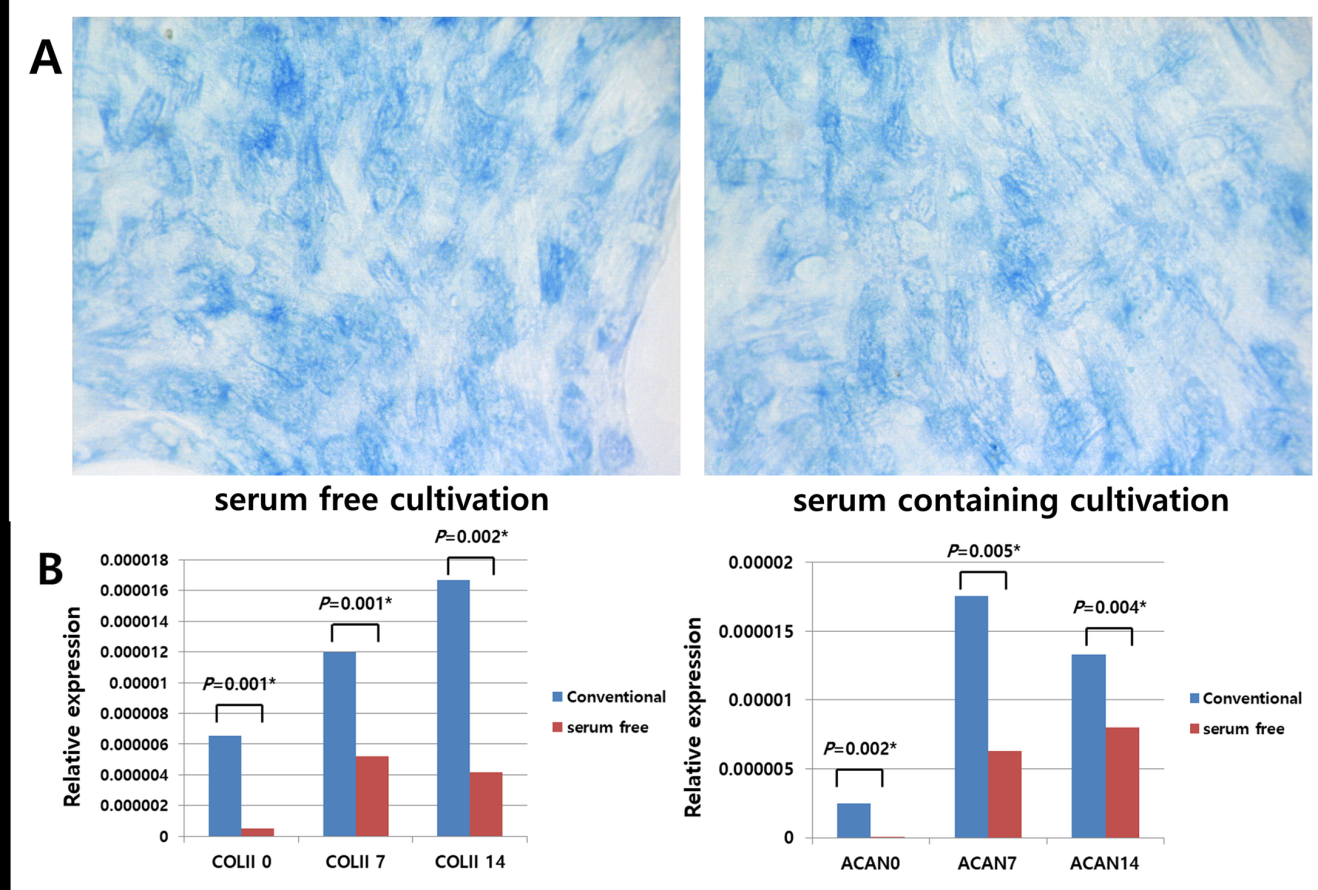
Comparison of the chondrogenic differentiation potentials of hTMSCs cultured in serum-free medium versus those of hTMSCs cultured in serum-containing medium. Under chondrogenic conditions (A), visual assessment revealed similar levels of toluidine blue staining (x 400) in hTMSCs cultured under serum-free medium (left) and hTMSCs cultured under serum-containing medium (right). However, RT-PCR analysis of type II collagen and aggrecan mRNA expression in hTMSCs cultured under serum-free medium showed consistently increased expression, but much lower expression of chondrogenic differentiation markers compared with the levels in hTMSCs cultured under serum-containing medium.

Under osteogenic conditions, ALP staining identified comparable amounts of calcium mineralization in the two groups (Fig 4A). Similarly, adipogenic conditions culture caused a considerable amassment of lipid droplets in the two groups, as detected by positive Oil Red O staining (Fig 5A). Under chondrogenic conditions, cells from both groups demonstrated the typical sulphated extracellular matrix, as revealed by toluidine blue staining (Fig 6A).

Additionally, RT-PCR was conducted to quantitatively analyze the manifestation of differentiation-related genes. Cells exposed to differentiation medium consistently displayed upregulation of mRNAs encoding osteocalcin, type I collagen, Runx2 (osteogenic), PPARr, and ACS (adipogenic), type II collagen, aggrecan (chondrogenic). However, significant differences in the differentiation capacities of the two hTMSC groups were observed. Specifically, type I collagen, Runx2, PPARr, and ACS were significantly upregulated in the SFXFM group versus the control group (Figs 4B and 5B). In contrast, type II collagen and aggrecan were significantly downregulated in the SFXFM group versus the control group (Fig 6B). These results demonstrate that the hTMSCs in the SFXFM group exhibit higher osteogenic and adipogenic, but not chondrogenic, differentiation potential.

### Genomic stability of expanded hTMSCs cultured in serum-free medium

**Fig 7:**
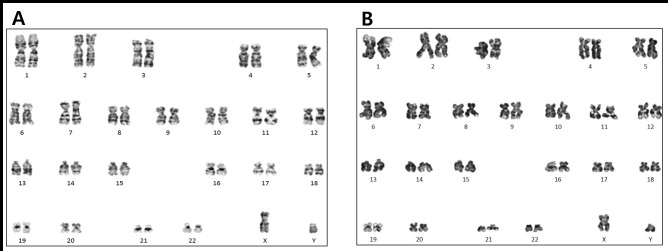
Karyotype analysis of hTMSCs cultured in serum-free medium. To check whether cells derived from serum-free cultivation showed the chromosomal stability or not, the cytogenetic karyotypes of cells at passage 3 (left) and 6 (right) were analyzed. All sex chromosomes were XY. No chromosome eliminations, displacements, or imbalances were observed.

To determine the impact of SFXFM on the genomic stability of the hTMSCs, we analyzed the karyotypes of SFXFM-cultured hTMSCs in metaphase at passage 3 and 6. No gross chromosomal aberrations were observed, suggesting that serum-free conditions preserved the normal cell karyotype (Fig 7), which could showed that cells would be expanded safely before clinical use to reach the required number.

## Discussion

Cell-based therapies typically require a huge amount of cells [8]. To satisfy this large need, efficient procedures related to the expansion of MSCs need to be in place before these therapies can achieve wide clinical use. In general, bovine serum-containing medium and porcine-derived trypsin have been applied to the MSC expansion [11]. However, in clinical cell therapies, the use of these components can induce a potential risk related to unknown antigens or microbes. Additionally, high levels of xenogeneic proteins raise the concern of immune reactions in human patients [11,12].

Recently, a number of serum-free or xeno-free media formulations regarding MSCs culture have been developed to achieve improved safety, purity, and potency [13]. Many commercial serum-free and xeno-free media formulations have been considered to be hopeful substitute for the production of MSCs for clinical use, since they have the potential to overcome the challenges posed by serum-containing media [13]. StemPro MSC-SFM was the commercially available SFM and FDA-approved xeno-free culture media for the expansion of BM-MSC and Ad-MSC for medical utilization [8]. Several studies have compared the characteristics of MSCs grown in serum-free StemPro-SFM-XF with those of MSCs grown in FBS-supplemented medium, with the aim of determining whether these MSCs are suitable for clinical applications [12–14].

Human MSCs are expected to demonstrate biological discrepancies that influence the functional potential in accordance with the tissue from which they are originated [2]. In our previous study, the cell yield was 6.55 x 10^3^ cells/milligram of turbinate, which showed approximately thirty times higher yield than adipose tissue (0.2–0.29 x 10^3^ cells/ milligram). In addition, it was proved that the hTMSCs exhibited approximately 5 times higher proliferation than the hBMSCs [15]. 3 Additionally, it has been shown that the donor age do not influence the properties of hTMSCs, unlike other human MSCs [4] and local condition of nasal cavity including allergic state or nasal septal deviation also do not have the effect on the feature of them [16,17]. The excellent potency of hTMSCs compared to MSCs from other tissues could make our group pay particular attention to the cells. We presumed that the potency of these hTMSCs facilitates the development of an effective method for tissue regeneration. However, it has become evident that these MSCs must be expanded in defined medium that is FBS-free for applications in clinical research. In this study, we isolated and expanded hTMSCs in StemPro-SFM-XF [11]. We compared the characterization, proliferation, and functional characteristics of hTMSCs with those of cells cultured under FBS-containing medium.

Specifically, we compared the characteristics of hTMSCs cultured in SFXFM with those of cells cultured in serum-containing media (n = 5 per group). In view of shape, cells grown under the two conditions maintained a similar size, and both exhibited the characteristic spindle-shaped appearance. Some researchers have shown that BM-MSCs and Ad-MSCs cultured under SFM display a smaller, narrower spindle-shape versus wide spindle-shaped cells cultured under FBS-containing medium [7,14]. By contrast, other studies have observed no differences with respect to size or shape between BM-MSCs grown under different culture conditions. However, Ad-MSCs grown in SFXFM did appear smaller in size compared to Ad-MSCs grown in FBS-containing medium [9]. These discrepancies may have arisen due to donor-to-donor variation or the subjective aspect of morphology evaluation.[18]. However, all MSCs cultured in serum-free media have been reported to maintain a fibroblastic-like morphology (i.e., adherent cells), despite these trivial morphologic differences. Additionally, in view of cell surface markers, the hTMSCs from both groups exhibited the characteristic MSC phenotype similarly. Considering the effects of culture media on our findings, the identical cell surface marker profile applies to hTMSCs cultured under serum-free conditions [7].

In cellular proliferation assay, hTMSCs cultured in the presence of serum tended to exhibit higher proliferation from days 3 to 4 than those cultured in serum-free medium. Nevertheless, no significant discrepancies of proliferation were shown in the two groups during the 7-day period except those of days 3 and 4, which are similar with our previous studies pointing out that hTMSCs exhibit a rapid growth pattern [4,5,19]. These findings suggest that the substitution of FBS by SFXFM can conserve the high proliferation potency of hTMSCs, thereby potentially reducing the MSC production time. However, this finding is in contrast to previous results showing that SFXFM significantly enhanced the proliferation capacity of MSCs compared with MSCs cultured in serum-containing media [7,11,14]. In contrast, it was reported that StemPro-SFM-XF did not escalate the proliferation rate of dental tissue-derived MSCs [8,20]. The contradictory results imply that the functional potentials of human MSCs are influenced by culture conditions according to the source from which they are derived; however, additional studies are necessary to further test this hypothesis.

During tri-lineage differentiation of hTMSCs, induced hTMSCs in both groups exhibited increased intensity of histologic staining. Visual assessment did not reveal any significant differences of these staining patterns between the two groups. Many trilineage-specific genes (type II collagen, aggrecan, Runx2, type I collagen, osteocalcin, PPARγ, and ACS) were constantly upregulated in both groups. However, the expression levels of these trilineage-specific genes were also significantly discrepant between the two groups. Specifically, osteocyte- and adipocyte-specific genes were upregulated in SFXFM-cultured hTMSCs versus hTMSCs cultured under conventional media; in contrast, chondrocyte-specific genes showed the reverse pattern. Agata et al. used StemPro MSC-SFM to induce differentiation into osteogenic lineages with BM-MSCs [8]. The in vivo osteogenic potentials of BM-MSCs were higher in BM-MSCs under StemPro MSC-SFM versus BM-MSCs grown in serum-containing medium. Moreover, serum-free cultured BM-MSCs demonstrated considerable osteogenesis (25.1%) versus serum-expanded BMMSCs (21.1%) [8]. Our results corroborate an earlier report that demonstrated strong osteogenesis potentials in xeno-free and serum-free cultures [8,21]. This finding highlights the potential use of hTMSCs in osteogenic applications. In contrast, the positive chondrogenic staining observed in our study (despite the low observed gene expression) is consistent with a previous study that found that serum-free cultured MSCs retained their chondrogenesis properties (as shown by histologic finding) [12]. Compared with controls, hTMSCs cultured under the serum free condition kept their differentiation capacity and could thus maintain the multipotency of the hTMSC population.

MSCs are well known to exert their immunomodulatory capacity via cell-cell contact or secretory components such as cytokines or chemokines [22]. Although comparison with other studies was difficult, since few studies have evaluating the cytokine secretion patterns of MSCs cultured under serum-free medium, we could make a few comparisons. For example, Wu et al. found that umbilical cord-derived MSCs cultured under serum-free media demonstrated strong immunosuppressive capacities and released similarly high levels of immunomodulatory cytokines such as IL-6 and IL-1β, but exhibited reduced mRNA expression of proinflammatory cytokines such as TNF-α, IFN-γ, IL-6, and IL-1β [23]. However, contrary to our expectations, culturing in SFXFM resulted in similarly high levels of IL-6 expression, upregulation of GM-CSF and IFN-γ, and downregulation of RANTES in hTMSCs compared with control cells. This discrepancy would be caused by disparity in the media or in the origin of MSCs. These results demonstrate that secretion of both proinflammatory and hematopoietic cytokines was increased in hTMSCs expanded in SFXFM. This finding indicates that serum-free medium includes strong inducers of cytokines that reduce the anti-inflammatory capacity of MSCs; these inducers could exhibit an accessional part by guarding the host from allogeneic defiance and protecting the host from its immunosuppressive properties [10,24]. However, the full extent of the immunomodulation controlled by MSC remains controversial. Further investigations in this area are needed to better understand this range.

Non-physiologic in vitro culture conditions could conceivably cause mutations and chromosomal aberrations. These chromosome variabilities have been related with the progression of malignant mesenchymal tumors [25]. Although most studies on chromosome variability have been performed on human MSCs derived from various tissues, discrepant results have been obtained regarding the accumulation of chromosomal aberrations during in vitro culture [26,27]. However, some studies observed spontaneous numerical and structural chromosome aberrations within 10 passages of in vitro culture [28,29]. The specific in vitro environmental conditions would be a major factor influencing the conservation of genome integrity during culture [30]. In this study, no clonal chromosomal aberrations were identified in hTMSCs cultured in SFXFM. The results resemble those of previous studies [11,31], implying that supplement origin does not play a role in the maintenance of genome stability [30].

We firstly identified that hTMSCs cultured in SFXFM manifest MSC-specific surface makers, proliferate highly, and show trilineage-differentiation. Considering the anticipated increase in demand for MSC-based cell therapies for future clinical application, our results suggest that hTMSCs cultured in xeno-free, serum-free medium are superior with respect to clinical use compared with autologous or allogenic hTMSCs. However, further evaluation of hTMSCs will be important for identifying new suitable sources of MSCs.

## Conclusion

Our data demonstrate that hTMSCs expanded in xeno-free, serum-free medium maintained all vital characteristics of MSCs for possible clinical application. This finding will facilitate the development of an efficient way for regenerative medicine due to the usability of a considerable amount of tissue that was removed. Raw date was presented in the S1 File.

## Supporting information

S1 File Raw data of cytokine level, gene expression level related to differentiation, and proliferation. The raw date regarding cytokine level, gene expression level related to differentiation, and proliferation was presented in the Excel file.
(XLSX)
